# Managing chronic hepatitis B: A qualitative study exploring the perspectives of people living with chronic hepatitis B in Australia

**DOI:** 10.1186/1756-0500-4-45

**Published:** 2011-03-03

**Authors:** Jack Wallace, Stephen McNally, Jacqui Richmond, Behzad Hajarizadeh, Marian Pitts

**Affiliations:** 1 Australian Research Centre in Sex, Health and Society, La Trobe University, 215 Franklin Street, Melbourne VIC, Australia; 2 St Vincent's Hospital, 41 Victoria Parade, Fitzroy VIC, Australia

## Abstract

**Background:**

The implementation of a comprehensive public health response to hepatitis B in Australia is urgently required to reduce the increasing burden of hepatitis B infection on the health system and the community. A significant gap in the public health response to hepatitis B is an understanding of how people with chronic hepatitis B (CHB) respond to CHB.

**Findings:**

A qualitative study involving semi-structured interviews and focus group discussions was conducted. Interviews were held with 20 people with CHB from three states of Australia. In addition, four focus group discussions were held with a total of 40 community and health workers from culturally and linguistically diverse communities in four Australian states.

People with CHB reported no formal or informal pre or post test discussion with little information about hepatitis B provided at the point of diagnosis. Knowledge deficits about hepatitis B were found among most participants. Few resources are available for people with CHB or their families to assist them in understanding the infection and promoting their health and well-being. A lack of confidence in the professional knowledge of service providers was noted throughout interviews.

**Conclusions:**

People with CHB need culturally and linguistically appropriate education and information, particularly at the point of diagnosis. Primary health care professionals need the knowledge, skills and motivation to provide appropriate information to people with CHB, to ensure they have the capacity to better manage their infection.

## Background

Hepatitis B is a blood-borne and sexually transmitted viral infection. More than 2 billion people globally have been exposed to hepatitis B with an estimated 350 million people being chronically infected. Hepatitis B is the cause of up to 1.2 million deaths per year worldwide [1].

Epidemiological studies estimate chronic hepatitis B (CHB) prevalence in Australia to range between 0.5 and 2.1 percent [2-4] with the differences related to indirect estimates and updated migration data. Most people with chronic hepatitis B in Australia were born in highly endemic countries in the Asia-Pacific region [3,5]. Despite improvements in clinical management, it has been estimated that only a small proportion of people with CHB, for whom treatment is recommended, receive antiviral therapy [3,6]. This is resulting in an estimated two to three fold increase in hepatitis B-related liver cancer cases, with the numbers of people dying as a result of CHB in Australia estimated to increase from 450 in 2008 to 1,550 in 2017 [7,8].

Previous health responses to hepatitis B in Australia have relied on vaccination, securing the blood supply and access to pharmaceutical treatments. This approach is inadequate in responding to the increasing burden of hepatitis B infection. The recent development of the National Hepatitis B Strategy 2010-2013 [9] with its goals of reducing the transmission, morbidity and mortality caused by hepatitis B, and to minimise the personal and social impact of hepatitis B provides the framework for implementing this response.

A significant gap in implementing this strategy is identifying how people with CHB respond to their infection. This study aimed to record this information by interviewing people with CHB and other stakeholders. The reported findings are one component of an extended qualitative research project aimed at developing a comprehensive public health response to hepatitis B in Australia.

## Methods

This qualitative study used interviews and focus group discussions given the absence of non-statistical research on people with CHB in Australia.

Face-to-face semi-structured interviews were carried out with 20 people with CHB from Victoria, New South Wales (NSW) and South Australia. Participants were recruited through a public hospital liver clinic in Victoria; non-government organisations including the Hepatitis C Council of South Australia; Hepatitis NSW; the Hepatitis Council of Queensland, and through the professional networks of three investigators who had been involved in national responses to other blood borne viruses.

Participants included 13 men and seven women with ages ranging from late teens to mid 60 s. Six participants were born in Vietnam; five in China, three in Cambodia and two from Afghanistan. Other participants were from Australia, North America, Greece, and Turkey. The majority of participants had been diagnosed in Australia within the previous 10 years with one person diagnosed six weeks prior to the interview and two participants diagnosed for 20 years. Purposive sampling ensured variability in age, gender, ethnicity, state of residence, and exposure route.

One researcher conducted all interviews, guided by a pilot tested interview schedule. The schedule sought information on how people were diagnosed; their clinical management experience, and their perspective on the effect on their lives of having hepatitis B. All but one interview was conducted in English with interpreters available if required.

Four focus group discussions were held in Victoria, NSW, South Australia and Queensland with staff and volunteers of non-government organisations providing health and social support services to the communities most at risk of CHB infection. Forty community or health workers working with 12 different culturally and linguistically diverse background (CALD) communities provided an understanding of the broader social context for individuals with CHB, and of the impact of hepatitis B at an individual, community and health service level. Focus group participants were recruited through the Ethnic Communities Council of Queensland, Multicultural HIV/AIDS and Hepatitis C Service in NSW, the Hepatitis C Council of South Australia and the Australian Research Centre in Sex, Health and Society in Victoria. The focus group discussion schedule, informed by the outcomes from interviews with individuals, sought information about community wide responses to hepatitis B; perspectives of the needs of people with CHB, and how these needs could be addressed; the importance of hepatitis B within communities, and the priority of hepatitis B to the communities most at risk. Focus group discussions took between 60 and 90 minutes.

All interviews and focus groups were recorded, transcribed, and verified. Data from the transcripts were coded using Nvivo8 (QSR International Pty Ltd, VIC, Australia). Analysis was conducted using grounded theory by organising data into codes from which main themes were identified as interviews progressed [10]. Two researchers independently analysed and discussed results to reach consensus. Focus group and interview data were compared, ensuring data and methodological triangulation. Saturation was reached by the end of interviews and focus group discussions.

The proposal was approved by the La Trobe University Human Research Ethics Committee and the Southern Health Human Research Ethics Committee (Victoria).

## Major themes

Major issues identified through the interviews and focus group discussion included testing and diagnosing of CHB; disclosure; clinical management, and participants' beliefs and concerns about chronic hepatitis B infection. All quotations are from people with CHB except where otherwise indicated.

### Testing and diagnosing infection

Hepatitis B testing occurred for participants in a range of settings and within differing life contexts. These included routine testing or as a consequence of employment screening; upon entry to drug rehabilitation, or as one part of the immigration process. For several participants, testing for CHB occurred within a context of entire families being tested:

My mother has hepatitis B ... and the family doctor [suggested the family] have [a] hepatitis B blood test ... then we all found out we have hepatitis B ... the whole family. (Chinese born woman, mid 30's)

No participant reported receiving a formal or informal pre- or post-test discussion and several did not remember providing consent for testing:

I didn't ask for it, just through a normal blood test (Vietnamese born man, late 30s)

Little information about hepatitis B or its impact was provided to most of the participants at the point of diagnosis. One Chinese born man in his mid twenties diagnosed four years prior to the interview reported:

*They didn't give me any information ... all I know is that it was hepatitis B*.

A major impact of the lack of pre - and post-test discussion and/or informed consent meant that receiving a CHB diagnosis was described by several participants as 'shocking.' One participant who had been diagnosed in Australia in 2007 noted:

*The family doctor ... told me that I got it, it was shocking, because I don't really fully understand what hepatitis B is but I know that sometimes it can lead to serious problems. (Chinese born woman, early 20's)*.

People's limited information and understanding of hepatitis B at the time of diagnosis created considerable confusion and fear for their future. The following quotes come from a Vietnamese born woman in her early 40 s who had been diagnosed for 10 years, and a woman born in China who had been diagnosed for 3 to 4 years:

I think it was like a cancer or something. How long will I live?

Will I survive? Will I be able to have kids?

When relevant and accurate information is not provided at the point of diagnosis, some participants sought information that reflected their cultural understandings of health from within their communities and through the internet. For one twenty year old man, born in Afghanistan, and diagnosed in a refugee camp, the lack of relevant information at the point of diagnosis required further investigation:

*I wasn't sure what does it mean hepatitis B ... I asked some other people and then they say this means this kind of thing in our language we say ... it means that this skin is yellow*.

One health worker reported her experience in counselling a person with CHB that reflected the essential nature of pre/post-test discussion:

*He was an intelligent, educated young man. But because the GP hadn't told him or started that slow education counselling process, by the time he got to me it was a huge catastrophe, he was going to die, his wife was going to leave him... We need to situate the disease in all the cultural issues*.

## Disclosure

There was diversity among the participants' experiences in disclosing their infection to family and the broader community. The inter-generational nature of hepatitis B transmission for one young woman meant that there was no shame associated with infection, and that disclosing her hepatitis B status was straightforward.

I had nothing to hide because it was given to me from birth. (Australian born woman with Vietnamese parents, mid 20s)

However, disclosure was not easy for all people that we spoke with. Some respondents talked of complex rules about disclosure for different friends and family members in different situations. One man highlighted that shrewdness was required to determine to whom they would disclose their infection:

It's about being almost street-smart. (American born man, mid 40s)

One person had very clear rules about disclosure that reduced the risk of negative responses to their infection:

If they don't ask I'm not telling them. (Cambodian born man, late 30s)

However, the fear of rejection or stigma at a personal and community level was identified as an important issue that restricted individual disclosure:

I didn't let my brother know because he's only 10 ... I don't want him to ... go to school and tell anyone because kids are kids ... I don't want to change his life. (Chinese born woman, early 20s)

The impact of a CHB diagnosis and subsequent disclosure of this information within some communities was reported by a health worker to lead to exclusion from family and community life. Some community level responses highlighted established ways of dealing with vulnerability:

If you come from a different cultural background, the way you avoid disease is through exclusion, and so if that works with some things, then they [do the same with hepatitis] (Community Worker)

People are really fearful, so when one partner is diagnosed with hepatitis B ... the other partner is wanting to stay right away from them. (Community Worker)

Some of the health professionals noted less stigma related to hepatitis B particularly in comparison to other sexually transmitted diseases:

It's just called liver sickness ... hepatitis B is not seen as a sexually transmitted disease, because ... you don't get the genital symptoms, it doesn't have the stigma as syphilis. (Health Worker)

### Clinical Management

Perceptions of the availability, appropriateness and accuracy of information about hepatitis B for people with CHB and health professionals were key themes in participants' beliefs about good management for CHB.

Several people noted that their deficits in information had a significant effect on their behaviour which resulted in poorer health outcomes:

I thought it was only a weak sign so I didn't even bother about [treatment] ... so I didn't get regular blood tests or anything. (Chinese born man, early 20s)

If they told me a few years ago that I wasn't really meant to drink alcohol ... I would have cut down on it. (Cambodian born man, early 20s)

On the other hand, several participants reported effective health promoting responses such as reducing alcohol consumption:

After I found out I had hepatitis B, I quit drinking after they told me drinking could make it really worse for the liver .... It makes it better for life anyway. (Chinese born man, mid 20s)

Three people including, one who had been diagnosed in the past two years, noted that they weren't being treated for hepatitis B because: *not yet ...just a carrier. (Vietnamese born man, late 20s)*

In spite of Australia's policy of vaccinating all newly born children and providing vaccine and immunoglobulin to neonates born to hepatitis B infected mothers, missed opportunities for reducing transmission were noted by one respondent whose child was infected with hepatitis B at birth in Australia:

My daughter, she's only two and a half ... we just found out a year ago that she's got hepatitis B, she was born here. (Vietnamese born woman, mid 30s)

One participant, diagnosed six years previously, was concerned that his GP was unable to respond effectively to their need for information to help in self-monitoring:

I told the doctor that I had an e-antigen test, and he goes 'the result?' and I go 'I don't know, it's not active' or something and it was left there ... he hasn't followed [it] up. (Australian born man, late 20s)

One community worker noted the advice of their agency to people with CHB had changed over time, given the awareness of the agency in improvements in hepatitis B clinical management. They were concerned that GPs were not responding effectively to the needs of people with CHB:

*If you've been diagnosed with chronic hepatitis B, ask your GP for a referral to a specialist ... I've noticed that GPs will sit on hepatitis B in their own room for years and years*.

There were several instances when community and health workers disclosed their lack of knowledge about CHB:

Even the natural history - I'm not clear about it ... I don't even know if treatment is available (Community Worker)

### Knowledge, beliefs and concerns

Given the nature of CHB infection, several participants reported other family members were infected and that this affected family relationships. Sometimes these familial links, along with a lack of understanding of hepatitis B, led to assumptions that challenged the view of hepatitis B as a virus:

*The whole family have hep B except for my father ... maybe it's a blood thing. (Cambodian born man, late 30s)*.

Some people diagnosed with CHB in their country of origin, reflected on the endemic nature of hepatitis B in that country, and the lack of options available:

I got a test in Cambodia and the doctor told me I have hep B, but in Cambodia it's like normal, they don't care what happens. (Cambodian born man, late 30s)

Many participants associated hepatitis B with poor sanitation, reflecting an inadequate understanding of transmission risks:

I think I was infected in China, and we used to share foods, drinks, so I think that's how I got infected. (Chinese born man, early 20s)

A community worker working with people from CALD backgrounds who inject drugs noted a perspective indicating a relationship between hepatitis and HIV, with people progressing: *from hepatitis A, to hepatitis B, and hepatitis C then to HIV*.

This incorrect understanding of viral hepatitis was also noted by one man with CHB who understood hepatitis as:

Three levels - hepatitis A, hepatitis B and hepatitis C with hepatitis C being worse. (Afghani born man, mid 50s)

## Discussion

This study provides insight into the understandings and responses to CHB infection by individuals with CHB. The study found that most participants had not consented to testing and received little or no information at diagnosis; people with CHB had a poor understanding of their infection, and had reservations in the capacity of health professionals to respond effectively to CHB.

For most of our participants, testing for CHB infection occurred without the explicit informed consent of the patient, and the CHB diagnosis occurred without either written or verbal information being provided to assist the patient understand the social implications or clinical impact of the infection. National hepatitis C and HIV testing policies have been developed in Australia for health care workers including general practitioners (GPs) that detail best practice in testing and diagnosis, both of which are described as: 'essential for a patient to make an informed decision regarding ... testing' [11]. Both the HIV and hepatitis C testing policies specify pre-test and post-test discussion processes including ensuring that the patient provides consent for the test, that the patient is provided with information about the social and clinical implications of diagnosis, including information about the condition being tested for, clinical management options, and health promotion information. While a testing protocol for hepatitis B has been identified [5], its development and implementation has not yet received national recognition or endorsement. Evidence based counselling guidelines would provide a framework for ensuring that people with hepatitis B understand the disease and prognosis and are able to appropriately respond to their infection [12].

The majority of people with CHB in Australia come from CALD backgrounds, primarily from the Asia Pacific region, and it has been previously reported that these populations have inadequate levels of awareness and knowledge about hepatitis B (13-15). The qualitative methodology used in this study supported the findings of these studies and provided a more nuanced perspective on the confusion about relationships between hepatitis viruses, and hepatitis viruses and HIV, treatment and transmission risks.

Chronic hepatitis B is a complex condition mostly affecting people who may have understandings of the body that differ from those of the western medical model. Providing information to people with CHB describing the implications of infection requires a comprehensive understanding of the virus, and the skills to be able to impart this information in a clear manner. People with CHB expressed a lack of confidence in the professional knowledge of service providers. General practitioners and other primary health care professionals need to be equipped with the information, expertise and motivation to ensure that people with CHB understand the natural history of the virus, the rationale for clinical monitoring and the available treatments. The term 'carrier' with an assumed meaning of chronic hepatitis B being inactive is not supported by current clinical management and yet was misunderstood by participants as an encouraging sign. The lack of relevant information provided to, or recalled by people with CHB about their infection provided opportunities for people to seek explanations of their infection that were culturally relevant, although not necessarily accurate. Our findings show that understandings of hepatitis B may be influenced by cultural background, and that culturally and linguistically appropriate interventions are required to improve knowledge and assist in providing higher standards of care [16,17].

Australian public health responses to HIV and hepatitis C acknowledge the role of stigma and discrimination as a result of transmission occurring through sex or injecting drug use in the majority of infections [18,19]. Some participants in our study reported selective disclosure of their infection to other people. Despite particular cultural beliefs among some CALD communities about illness or blood borne viruses, we found less evident stigma related to hepatitis B than for hepatitis C and HIV [20], particularly given the familial nature of infection for many of our participants. This is an area in which further research is required.

Given sampling limitations, these findings have limited generalisability; nonetheless, as the first qualitative study exploring the perspective of people with CHB in Australia, this study provided information for the development of a public health response to hepatitis B. In addition, the qualitative data describing hepatitis B knowledge, beliefs, and behaviours of people with CHB can be used to develop population-based surveys. Such surveys could quantify the knowledge and attitudes of people with CHB and be used to develop culturally appropriate community-based interventions.

English language proficiency was identified as the main barrier in communicating between health care providers about CHB in a study of Asian-Americans [21]. This barrier was not directly addressed in our study where most interviews were held in English and participants had at least minimal English proficiency.

GPs were not specifically targeted for interview in this study. Qualitative research describing GPs knowledge and practice in response to CHB is a priority to identify their perspective on barriers to effective CHB management.

In conclusion, this study was the first in Australia and one of the few internationally [12,22], to provide qualitative data describing how people with CHB respond to their infection. This information included identifying significant gaps in culturally and linguistically appropriate information and education, particularly at the point of diagnosis. This provision of information will assist in improving the knowledge and understanding of people with CHB about their infection, and assist them in effectively managing this infection. Primary health care professionals, including GPs need the knowledge, resources, expertise and skills to provide relevant and accurate information to people with CHB. The role of stigma and discrimination, a key topic in Australian national responses to other blood borne viruses such as hepatitis C and HIV, needs further investigation to determine the extent and impact of these issues on people with CHB. The study provided data that informed the development of Australia's first National Hepatitis B Strategy.

## Competing interests

The authors declare that they have no competing interests.

## Authors' contributions

JW, SM and JR designed all aspects of the study, developed the initial research proposal, obtained funding, and analyzed findings. JW interviewed all participants with CHB; assisted in focus group facilitation and analysis, and prepared the first draft of the report. SM assisted in focus group facilitation and analysis. BH and MP analyzed study findings and prepared the first draft of this manuscript. All authors contributed to interpretation of results, have read and approved the final manuscript.
